# Follicular Regulatory T Cells in Systemic Lupus Erythematosus

**DOI:** 10.1155/2021/9943743

**Published:** 2021-07-13

**Authors:** Xin Xia, Jun Yang, Shengjun Wang

**Affiliations:** ^1^Department of Laboratory Medicine, The Affiliated People's Hospital, Jiangsu University, Zhenjiang, China; ^2^Department of Immunology, Jiangsu Key Laboratory of Laboratory Medicine, School of Medicine, Jiangsu University, Zhenjiang, China

## Abstract

Follicular regulatory T (Tfr) cells are the regulatory T cell subset mainly localized in the germinal center (GC), acting as modulators of GC responses. They can disrupt Tfh cell- and B cell-linked recognition, induce Tfh apoptosis, and suppress B cell function. Evidences show that dysregulated Tfr cells are associated with the disease activity index and serum autoantibody levels, influencing the development of systemic lupus erythematosus (SLE). This review focuses on the interaction among Tfr, Tfh, and B cells, summarizes the characterization and function of Tfr cells, concludes the imbalance of CD4^+^T subsets in SLE, and presents potential therapies for SLE. In general, we discuss the roles of Tfr cells in the progress of SLE and provide potential treatments.

## 1. Introduction

Systemic lupus erythematosus (SLE) is a chronic autoimmune disease characterized by red speckles on the skin and multiple organ damage. Current studies have revealed that imbalance of T helper (Th) cell subsets and regulatory T (Treg) cell subsets may contribute to the pathogenesis of SLE [1]. Previous studies showed that there were increased peripheral follicular helper T (Tfh) cells in autoimmune and inflammation diseases [2, 3]. Follicular regulatory T (Tfr) cells are the regulatory T cell subset mainly localized in germinal centers (GCs). It is suggested that Tfr cells can mediate Tfh and B cell reactions for proper GC responses [4]. The alteration of Tfh/Tfr ratios and frequency of Tfr cells were well correlated with the disease activity index and/or serum autoantibody levels [5]. Here, we discuss the features and roles of Tfr cells in SLE.

## 2. Characterization of Tfr Cells

Tfr cells are first proposed as an effector Treg subset derived from natural regulatory T (nTreg) cells. They were usually described as CD4^+^CXCR5^+^CD45RA^lo^CD25^hi^CD127^lo^ cells [6, 7]. Based on RNA-sequencing analysis (RNA-Seq), this subset not only shared phenotypic characteristics with Treg such as FoxP3, CTLA-4, GITR and Blimp1 but also had Treg-associated genes such as *FoxP3*, *Ctla4*, *Gitr*, and *Prdm1* [8]. They also expressed Bcl-6, CXCR5, PD-1, and ICOS, which was similar to Tfh, and had Tfh-associated genes such as *Bcl6*, *Cxcr5*, *Pdcd1*, and *Icos*.

Previously, the majority of Tfr cells resident in the mouse secondary lymph tissues were thought of as CD25^+^ Tfr cells. Some studies found that there were CD25^−^ Tfr cells [8–12]. CD4^+^PD-1^+^CXCR5^+^FoxP3^+^CD25^−^ cells can be divided into Tfr cells. It was reported that based on the single-cell transcriptomics Tfr cells lost CD25 expression as they matured [12]. Besides, there were almost CD25^−^ Tfr cells in mouse GCs [8]. After immunization, CD25^−^ Tfr cells were preferentially located in GCs. CD25^+^ Tfr cells maintained after the peak of GC reaction [13]. It is suggested that CD25^−^ Tfr cells but not CD25^+^ Tfr cells are the effector cells in GC responses. Due to their different stages of development having their corresponding locations, it is considered that Tfr markers change during their multistep differentiation processes. We can describe them based on their locations [7, 14]. In the T cell zones, they are CD25^+^FoxP3^+^CXCR5^+^. In the follicles, they are CD25^-/+^FoxP3^+^CXCR5^+^Bcl-6^+^PD-1^+^ICOS^+^. In the GCs, they have similar phenotypes presented in the follicle, but the intensities of CXCR5, Bcl-6, PD-1, and ICOS are much higher in the GCs. In the circulation, they are CD25^+^FoxP3^+^CXCR5^+^.

There were differences between mice and men. It was reported that different independent tissues had distinct proportions of follicular T cells in different maturation states [12]. The ratios of CD25^−^ Tfr cells varied in human secondary lymph tissues. For example, there were rare Bcl6^hi^CXCR5^hi^PD-1^hi^Helio^+^FoxP3^+^CD25^−^ Tfr cells in the lymph nodes [15], but there were nearly only CD25^−^FoxP3^+^Bcl6^hi^CXCR5^hi^PD-1^hi^ Tfr cells in the tonsils. Besides, the level of CD25 expression was greatly reduced on activation [8]. The reduction of CD25 expression led to lower responsiveness to IL-2, which had a negative impact on the proliferation of Tfr cells [16]. With further investigation of human mesenteric lymph nodes, they found that most Tfr cells expressed low-to-intermediate level of PD-1 and resided at the T-B and GC-mantle borders. Tfr cells expressing a high level of PD-1 were mainly distributed in GCs [15].

## 3. Circulating Tfr Cells

**Figure 1:**
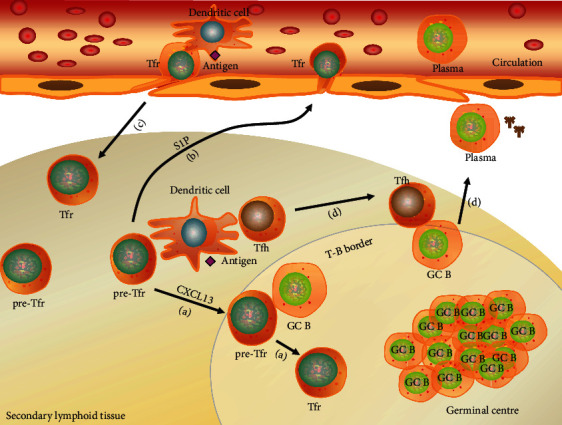
Circulation of Tfr cells. (a) In the secondary lymphoid tissue, after primary antigen presentation by DC, the pre-Tfr cell expressing high CXCR5 and CD69 follows CXCL13 gradients from the T cell zone to the B cell zone. Then, the pre-Tfr cell interacts with GC B cell to strengthen the Tfr cell signatures. (b) After primary antigen presentation by DC, the pre-Tfr cell expressing low-to-intermediate CXCR5 and low CD69 exits secondary lymphoid tissue through S1P gradients and enters the circulation. (c) In the circulation, the Tfr cell returns to secondary lymphoid tissues upon secondary antigen presentation by DC. (d) After DC presents the antigen, Tfh cell is activated to promote GC B cell to mature.

In the mouse secondary lymphoid organs, such as the lymph node and spleen, follicular CD4^+^FoxP3^+^ cells derived from the thymus can differentiate into Tfr cells, in response to primary antigen presentation by DCs [17, 18]. There is a model of initial Tfr cell differentiation (Figure 1). With the help of EBI2 (GPR183) [15], the Tfr cells expressing high CXCR5 and CD69 migrate to the T-B border, then follow CXCL13 gradients to the B cell zone. They are able to function as effector GC-Tfr cells after differentiation. In the B cell zone, Tfr cells experience the second phase of differentiation. After interacting with B cells, the Tfr signatures are enhanced. Besides, the Tfr cells expressing low-to-intermediate CXCR5 and low CD69 exit lymph nodes through sphingosine-1-phosphate (S1P) gradients and enter the circulation. The study showed that circulating Tfr cells nearly had no suppressive function, but they had memory-like features [19]. It means that they can recirculate from the blood upon secondary antigen presentation by DCs and return to the secondary lymphoid organs or tissues quickly in few hours in order to exert suppressive function. This model is further supported by the study that the location of Tfr cells was correlated with the expression of CXCR5 [11].

In the human tonsil, CD69^−^ Treg cells upregulated CXCR5 to migrate towards CXCL13-enriched GC. It is also reported that human circulating CXCR5^+^ Treg cells were Tfr cell precursors that emerged after birth from lymphoid tissues as immature cells endowed with partial humoral regulatory function [16]. Recent study found that the main maturation of Tfr cells followed a bifurcated trajectory from precursor Treg cells, with one arm of the bifurcation leading to blood Tfr cells and the other leading to the most mature GC Tfr cells [12]. Although most Tfr cells in the blood expressed CD45RA, they were able to be recruited to CXCL13-enriched tissues. It is suggested that mature Tfr cells primarily arise independently from their less mature counterpart in blood.

## 4. Function of Tfr Cells

Previous studies have inferred the function of Tfr cells. Tfr cells can inhibit IL-21 and IL-4 expressions by Tfh cells [20]. They can also control the activity of GC B cells and depress the initial activation of B cells through epigenetic changes and multiple pathways. Meanwhile, Tfr cells can suppress B cell downstream effector responses, such as class switch recombination, antibody production, and somatic hypermutation [11, 21].

**Figure 2:**
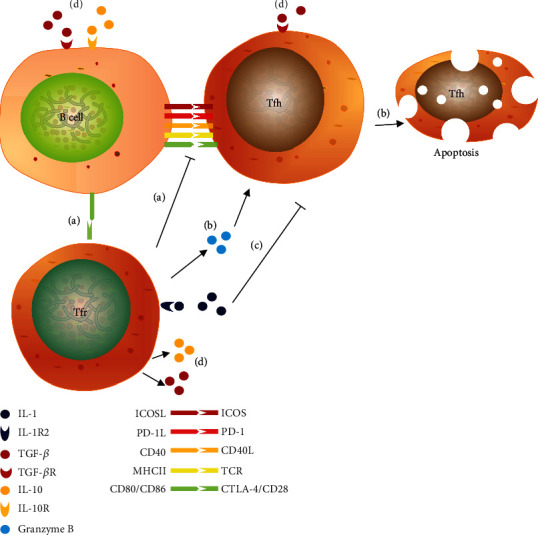
Function of Tfr cells. (a) Tfr cells can inhibit the physical interaction between Tfh and B cells by combining CD80/CD86 in B cells through CTLA-4. (b) Tfr cells can secrete granzyme B to induce the apoptosis of Tfh cells. (c) Tfr can express IL-1 decoy receptor IL-1R2 to engage IL-1, preventing IL-1 from interacting with Tfh cells. (d) Tfr cells can secrete IL-10 or TGF-*β*. The cytokines combine their receptors, respectively, stimulating downstream signaling pathways.

Even though the whole mechanism of Tfr suppressive function has not been thoroughly elucidated, some ways have been explained (Figure 2). Tfr cells can secrete IL-10 or TGF-*β* to regulate the GC reaction. IL-10 can impede GC responses and humoral immunity. It was also found that IL-10 secreted by Tfr cells promoted B cell differentiation and GC responses through inducing nuclear FOXO1 translocation in activated B cells, which contributed to the dark zone phenotype and affinity maturation during acute viral infection [22]. It means that IL-10 plays a multifaceted role in Tfr-mediated GC responses. TGF-*β* can suppress Tfh function and prevent Tfh cell accumulation. Besides, it can prevent self-reactive B cell activation and autoantibody production [23]. In addition, Tfr cells can disrupt the physical interaction between Tfh and B cells, inhibiting B cells from producing antibodies [18, 24]. Some evidences proved that there were rich GARP on Tfr surface [15]. GARP can support TGF-*β* to anchor Tfr cells. Besides, Tfr cells can combine CD80/CD86 in B cells through CTLA-4 in themselves and further secrete granzyme B to induce the apoptosis of Tfh cells [25]. Nonetheless, B cells will undergo another way of somatic hypermutation (SHM) [25]. In addition, it was found that murine Tfr cells prevented IL-1 from interacting with Tfh cells through expressing IL-1R2 [9]. IL-1R2 is a decoy receptor to engage IL-1. IL-1 can control Tfh cell activation and influence antibody production.

The ways how Tfr cells work are also supported by the observations in genetic studies including RNA-Seq and single-sample gene set enrichment analysis (GSEA) [21]. There were substantial changes in metabolism pathways in both Tfh cells and B cells. Firstly, the expressions of their effector molecules were actively downregulated. After being suppressed by Tfr cells, Tfh cells retained a unique state that did not strongly resemble anergy and exhaustion. The expressions of *Prdm1*, *IL-4*, and *IL-21* were reduced in Tfh cells, while *Cxcr5* expression was slightly increased. Suppressed B cells had lower expressions of *Ighg1*, *Ighg2c*, and *Igha*. Three function-associated genes *Pou2af1*, *Xbp1*, and *Aicda* were most attenuated in B cells, which was responsible for the durability of Tfr-mediated suppression. Secondly, Myc pathways were restrained in B cells suppressed by Tfr cells. The lower expressions of most genes targeting Myc had a similar extent to the level achieved with Myc inhibitors. Overexpression of Myc partially restored the suppressive influence of Tfr cells in the aspect of B cell proliferation. Besides, the suppressed B cells showed a deficiency in metabolic and anabolic pathways [26].

## 5. Tfr Cells in SLE

Studies have confirmed that the imbalance of Th subsets and Treg subsets can contribute to the pathogenesis of autoimmune diseases [27]. There were increased Tfh cells and decreased Tfr cells in rheumatic diseases [28–31], myasthenia gravis (MS) [27], and multiple sclerosis. However, not all studies of Tfr cells in autoimmunity are consistent. There were increased Tfr cells in primary Sjögren's syndrome [32] and ankylosing spondylitis [13, 14].

SLE is a chronic systemic inflammatory autoimmune disease with clinical symptoms varying from skin lupus erythematosus, such as cutaneous lupus erythematosus (CLE), to systemic diseases, such as lupus nephritis (LN). There was a great decrease of lymphocytes in SLE patients. As shown in Table 1, the imbalance of CD4^+^ T cell subsets is regarded as an important factor to the pathology of SLE [33].

### 5.1. SLE

The frequency of Tfh (CD4^+^CD25^−^CD127^int-hi^CXCR5^+^) cells was higher in SLE peripheral blood compared to healthy controls, while the frequency of Tfr (CD4^+^CD25^+^CD127^lo-int^CXCR5^+^) cells was lower in SLE patients [34]. In addition, the Tfh/Tfr ratio was much higher in patients with SLE. This study further researched the correlations between the frequency of Tfr cells and the level of serum antibodies. It was reported that there was a negative correlation between Tfr cells and anti-dsDNA, and there was no correlation between Tfr cells and IgG. Additionally, there was an increased percentage of PD-1^hi^ICOS^hi^Ki-67^+^ Tfr cells in SLE, which indicated the activation of Tfr cells in SLE. And the coculture experiments with memory B cells in vitro revealed that the suppressive capacity of circulating Tfr cells did not change in SLE patients [35]. Interestingly, SLE disease activity measured by the SLE disease activity index (SLEDAI) was not correlated with Tfh cells. However, there was a negative correlation between SLEDAI scores and Tfr cells and a positive correlation between SLEDAI scores and Tfh/Tfr ratio. When further investigating the relationship between SLEDAI and Tfr cells, it turned out that the patients with low disease activity (SLEDAI = 0-4) had higher Tfr percentage and lower Tfh/Tfr ratio than the patients with active disease activity (SLEDAI > 4). There was no significant difference of Tfh cells in SLE patients with different SLEDAI scores.

When the Tfr cells were represented as CD4^+^CXCR5^+^FoxP3^+^ and the Tfh cells were described as CD4^+^CXCR5^+^FoxP3^−^, there was a totally different result [35]. Both the percentage and frequency of Tfr cells were significantly elevated in SLE peripheral blood compared to healthy controls. Tfh cells were increased in SLE as well. But the Tfh/Tfr ratio was much lower in patients with SLE. There was a greatly positive correlation between Tfr cells and serum IgG, and there was no significant correlation between Tfr cells and anti-dsDNA. In the progress of studying the relationship between Tfr cells and disease activity, it was found that there was a positive correlation between CD4^+^CXCR5^+^ cells and SLEDAI scores, and the Tfr/Tfh ratios were positively correlated with SLEDAI scores. When sorting Tfr cells, CD127^lo^CD25^+^ can be used in placed of FoxP3 [36]. However, it is possible to contain Treg cells with gating CD25^+^ cells. These Tfr cells may be Treg cells with demethylation of FoxP3 CNS2 [8, 9].

### 5.2. Lupus Nephritis

Clinically, the majority of SLE patients suffer from kidney damage. Proinflammatory factors can cause kidney damage through the Jak2-Stat3 pathway. LN is considered a relevant feature of childhood systemic lupus erythematosus, accounting for 10-15% of all SLE cases. Previous studies have implied that Treg (CD4^+^CD25^hi^FoxP3^+^) cells were decreased in LN patients and negatively correlated with SLEDAI scores [37, 38]. It was also reported that circulating Th17 cells was increased in LN compared to healthy controls, but Treg cells remained unchanged [39]. Tfh cells were found increased in the spleen of MRL/Ipr mice. The percentage of Tfr (CD4^+^CXCR5^+^FoxP3^+^) cells was lower in 16-week-old MRL/Ipr mice than in 12-week-old MRL/Ipr mice [38]. Lupus-prone mice on the onset stage of LN had more Tfr cells than those on the end stage, indicating that the progress of lupus autoimmunity was correlated with a decline of Tfr cells.

### 5.3. Cutaneous Lupus Erythematosus

Skin disease is the second manifestation which can appear in any stage of SLE. CLE is a characterized by photosensitivity, apoptosis of keratinocytes, and an inflammatory infiltrate in the skin. Discoid lupus erythematosus (DLE) and malar rash are the categories of acute and chronic CLE, respectively.

DLE is graded to DLE-I, DLE-I/S, and DLE-S by dermal scarring, alopecia, and dyspigmentation. In DLE skin, the abundance of different CD4^+^T subsets varied in different disease phases. In early lesions, there were higher percentages of Th1 cells than Th2 cells in the perivascular and interface regions. In mid-stage and end-stage lesions, there were increased Th2 cells in the perivascular and interface regions [40]. And there were higher percentages of CD4^+^FoxP3^+^ cells in all stages [41]. Even though Th17 cells are thought of as an important pathogenic factor in SLE, few Th17 cells were found in DLE skin [42].

Malar rash patients had higher levels of IL-6 and IL-17 compared to healthy controls [43]. It was found that Th17 cells and Treg cells were imbalanced, and the serum levels of inflammatory cytokines such as IL-6 and IL-17 were greatly higher. In onset SLE patients, patients with malar rash had lower percentage of Th1 cells and relatively higher percentage of Th2 cells [44].

### 5.4. Neuropsychiatric Systemic Lupus Erythematosus

Neuropsychiatric systemic lupus erythematosus (NPSLE) is the manifestation involved in the nervous system and psychiatric disorders in SLE patients. In NPSLE, Th1 cells were increased [45]. They can produce IFN-*γ* and TNF-*α* to stimulate CXCL10 secretion from other cells and perpetuate disease process [46]. Tregs (CD4^+^CD25^hi^FoxP3^+^) were greatly reduced in patients compared to health controls [37]. The percentages of Treg were correlated with SLEDAI scores.

## 6. SLE Cytokine Profiles

Even though the autoantibodies of SLE target at a broad range of self-antigens, especially nuclear components. The characteristic autoantibodies are anti-Sm and anti-dsDNA antibodies which predominately perturb the function of multiple organs and systems. SLE patients have unique cytokine secretion profiles. There were decreased serum levels of TGF-*β* and IL-2 with a slight reduction of Treg in SLE patients [49]. TGF-*β* can prevent Tfh accumulation and induce Tfh apoptosis. They can also suppress B cell activities including survival, proliferation, differentiation, and antibody secretion [23]. Besides, TGF-*β* can induce FoxP3 which is essential for the development of Tregs in the periphery. IL-2 can induce conversion of memory Tfh cells to functional Tfr cells. It was through making STAT3 and STAT5 selectively bond to *FoxP3* and *Bcl6* gene loci, which was accompanied by suppression of H3K27me3 [50]. Blockade of both TGF-*β* and IL-2 signaling impeded Tfr development [51].

In addition, there were increased levels of serum IFN-*γ*, IL-21, IL-6, TNF-*α*, IL-17, IL-12, and IL-10 in SLE patients with positively comparable numbers of Tfh cells [52–54]. The secretions of IL-6, IL-10, and IL-17 were associated with global disease activity. It was reported that IL-21 and IL-6 promoted Bcl-6 expression via STAT1 and STAT3 signaling. Bcl-6 can induce CD4^+^T cells to express CXCR5, PD-1, ICOS, and CD40L. Bcl-6 and Blimp-1 are antagonistic regulators of Tfh and Tfr cell development [55]. IFN-*γ* and IL-12 can drive the differentiation program towards Th1 cell phenotype [56]. High production of IL-10 contributed to immunologic imbalance such as B lymphocyte hyperactivity and apoptosis of CD4^+^ and CD8^+^ T cells, which was related with disease activity and anti-dsDNA antibody.

## 7. Potential Therapeutic Target in SLE

Therapy keyed to specific cytokines or immunoregulators provides new strategies for SLE [14, 57]. Low-dose IL-2 treatment modulated homeostasis of Treg, Tfh, and Th17 cells in SLE patients along with great reductions of disease severity [58]. It is suggested that IL-2 can enhance the suppressive function of Treg cells and ameliorate Tfh- and Th17-mediated pathology. IL-21 treatment alleviated lupus-prone mouse symptoms. Overproduction of Tfh-promoting cytokines as well as TGF-*β*-rich circumstance promoted activated CD4^+^ T cells to downregulate Blimp-1 and express CXCR5, ICOS, IL-21, and IL-6. IL-21 and IL-6 exerted inhibitory function on Tfr-mediated suppression of metabolism and restoration of class switch recombination [59, 60]. IL-21 can rescue glucose uptake and increase lactate production by epigenetic changes. IL-21 blockade by anti-IL-21R or IL-21R deficiency controlled spontaneous arthritis in K/BxN mice [29, 30]. Intravenous immunoglobulin modulated the differentiation of CD4^+^T cells in the collagen-induced arthritis (CIA) model and upregulated the expression of IL-10 [57]. It is expected to become a possible therapeutic approach to SLE.

Tfr cells serving as an immunoregulator for autoimmune disease therapy are capable of inhibiting initial GC formation. It was reported that GC Tfh cells had the ability to migrate between the GC and interfollicular regions without entering the circulation. CXCR5^−^ Treg cells were possibly positioned at the T-B border instead of getting into the B cell follicle. It is suggested that when Tfr cells move, they may inhibit Tfh-B cell interactions. Besides, enforced expression of CXCR5 on Treg cells induced themselves to be Tfr-like cells, which suppressed Tfh cell-medicated aberrant IgG production in vitro [61]. The chimeric antigen receptor (CAR) technique will make these engineered Tfr cells antigen-specific. It was also supported that Tfr cells repressed the production of anti-dsDNA IgA in the pristine-induced lupus model [11]. It means that transfusion of Tfr cells may be a possible therapy for SLE patients.

Besides, the human circulating Tfr maintenance can be independent of ongoing GC responses [62]. RTX is a monoclonal anti-CD20 antibody rituximab. After RTX treatment, there was no effect on Tfr and Tfh cell numbers. Using RTX to deplete B cells and rebuild GC responses may be taken into account in the treatment of SLE.

## 8. Conclusion

Tfr cells noted as a subset of Treg cells extend the suppressive function of Treg cells in GC. They play an essential role in remedying aberrant antibody production, somatic hypermutation, and class switch recombination. Previous studies have revealed that there were dramatically increased circulating Tfh cells; elevated levels of IFN-*γ*, IL-21, IL-6, TNF-*α*, IL-17, IL-12, and IL-10; and reduced expressions of TGF-*β* and IL-2 in the SLE patients. Recent studies reported increased Tfh cells and decreased Tfr cells in SLE. The alteration was correlated with the disease severity. However, the indistinct definition of Tfr cells makes the study more difficult. More stringent Tfr standards should be set based on the differentiation phases, activation situation, or locations.

Given that Tfr and Tfh cells are reciprocal and antagonistic regulators of GC responses, disruption of their balance can result in excessive antibody production and autoimmune diseases. Tfr cells provide a new venue for immune modulators of GC responses to control the pathogenic process of SLE. In-depth works are needed to figure out the molecular mechanism of GC responses and the ways of correcting the disordered Tfh/Tfr ratio.

## Figures and Tables

**Table 1:** Imbalance of CD4^+^T cell subsets in SLE.

Disease	Cell subset					
	Th1	Th2	Th17	Tfr	Tfh	Treg
SLE	↑CD3^+^CD4^+^CD14^−^IFN-*γ*^+^ [44]	↑CD3^+^CD4^+^CD14^−^IL-4^+^ [44]	↑CD3^+^CD4^+^CD161^+^IL-17A^+^ [44]	↓CD4^+^CD25^+^CD127^lo-int^CXCR5^+^ [34], ↑CD4^+^CXCR5^+^FoxP3^+^ [35]	↑CD4^+^CD25^−^CD127^int-hi^CXCR5^+^ [34] CD4^+^CXCR5^+^FoxP3^−^ [35]	↓CD4^+^CD25^+^FoxP3^+^ [33]
LN	↑CD3^+^CD4^+^IFN-*γ*^+^ [47]	↓CD4^+^CCR4^+^CD294^+^ [48]	↑CD3^+^CD4^+^IL-17A^+^ [39, 47]	↓CD4^+^CXCR5^+^FoxP3^+^ [38]	↑CD4^+^CXCR5^+^PD-1^+^ [38]	↓CD4^+^CD25^hi^FoxP3^+^ [37] CD4^+^CD25^+^FoxP3^+^ [38] - CD4^+^CD25^hi^CD127^lo^ [39]
DLE	↑CD3^+^CD4^+^CD14^−^IFN-*γ*^+^ [44] CD4^+^T-bet^+^ [40] CD4^+^IFN-*γ*^+^ [42]	↑CD3^+^CD4^+^CD14^−^IL-4^+^ [44] CD4^+^GATA3^+^ [40]	↓CD4^+^IL-17A^+^ [42] ↑CD3^+^CD4^+^CD161^+^IL-17A^+^ [44]			↓CD3^+^CD4^+^CD25^hi^FoxP3^+^ [41, 44]
Malar rash	↑CD3^+^CD4^+^CD14^−^IFN-*γ*^+^ [44]	↑CD3^+^CD4^+^CD14^−^IL-4^+^ [44]	↑CD3^+^CD4^+^CD161^+^IL-17A^+^ [43, 44]			↓CD3^+^CD4^+^CD25^hi^FoxP3^+^ [41, 44]
NPSLE	↑CD4^+^IFN-*γ*^+^ [45]					↓CD4^+^CD25^hi^FoxP3^+^ [37]

Note: -: no difference, ↓: decreased, ↑: increased, blank: no results.

## Data Availability

The data supporting the conclusions of this article are included in the article.
